# Assembly of Biomolecular Gigastructures and Visualization with the Vulkan Graphics API

**DOI:** 10.1021/acs.jcim.1c00743

**Published:** 2021-09-16

**Authors:** Kornel Ozvoldik, Thomas Stockner, Burkhard Rammner, Elmar Krieger

**Affiliations:** † YASARA Biosciences GmbH, Wagramer Str. 25/3/45, 1220 Vienna, Austria; ‡ Center for Physiology and Pharmacology, Institute of Pharmacology, Medical University of Vienna, Waehringerstr. 13A, 1090 Vienna, Austria; § Sciloop, Karl-Theodor-Strasse 20, 22765 Hamburg, Germany

## Abstract

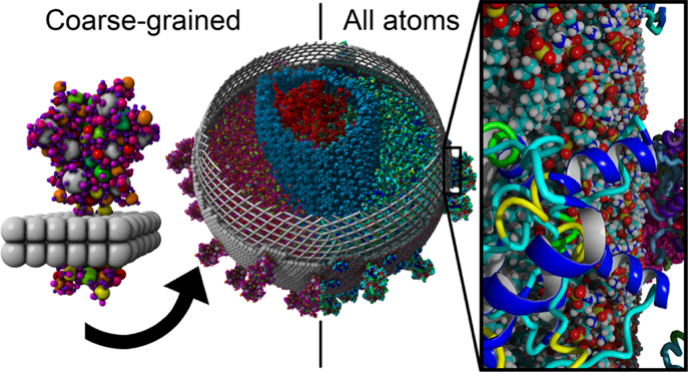

Building and displaying all-atom models of biomolecular structures with millions or billions of atoms, like virus particles or cells, remain a challenge due to the sheer size of the data, the required levels of automated building, and the visualization limits of today’s graphics hardware. Based on concepts introduced with the CellPack program, we report new algorithms to create such large-scale models using an intermediate coarse-grained “pet representation” of biomolecules with 1/10th the normal size. Pet atoms are placed such that they optimally trace the surface of the original molecule with just ∼1/50th the original atom number and are joined with covalent bonds. Molecular dynamics simulations of pet molecules allow for efficient packing optimization, as well as the generation of realistic DNA/RNA conformations. This pet world can be expanded back to the all-atom representation to be explored and visualized with full details. Essential for the efficient interactive visualization of gigastructures is the use of multiple levels of detail (LODs), where distant molecules are drawn with a heavily reduced polygon count. We present a grid-based algorithm to create such LODs for all common molecular graphics styles (including ball-and-sticks, ribbons, and cartoons) that do not require monochrome molecules to hide LOD transitions. As a practical application, we built all-atom models of SARS-CoV-2, HIV, and an entire presynaptic bouton with 1 μm diameter and 3.6 billion atoms, using modular building blocks to significantly reduce GPU memory requirements through instancing. We employ the Vulkan graphics API to maximize performance on consumer grade hardware and describe how to use the mmCIF format to efficiently store such giant models. An implementation is available as part of the YASARA molecular modeling and simulation program from www.YASARA.org. The free YASARA View program can be used to explore the presented models, which can be downloaded from www.YASARA.org/petworld, a Creative Commons platform for sharing giant biomolecular structures.

## Introduction

Experimental structure determination depends on the availability of large numbers of structurally identical copies that can be averaged, either in an X-ray crystal, in an NMR solution, or on a cryo-EM layer. On the mesoscale, where proteins, nucleic acids, and lipids assemble to form enveloped viruses, vesicles, bacterial cells, or eukaryotic cell compartments, the inherent randomness of the assembly processes makes sure that no two structures are the same. Electron microscopic images show a large variety of sizes and shapes, which preclude high-resolution structure determination. Alternatively, molecular modeling can serve as a route to fill these images with all-atom life, which is needed to improve our structural understanding of these large structures, to test hypotheses,^1^ and to create starting structures for simulations^2^ or for educational purposes.^3^ Two published approaches to mesoscale modeling are CellPack, which uses cooking recipes in XML or JSON format to describe how a model can be built automatically from its ingredients,^4^ and Marion, which takes an interactive approach and uses a graphical user interface to build a model with a set of rules.^5,6^

In this article, we describe a new functionality added to the YASARA molecular modeling and simulation program^7−9^ to construct mesoscale models from modular building blocks and visualize them using all common molecular graphics styles.

The model building process relies on an intermediate coarse-grained “pet world” representation whenever all-atom details would be computationally too costly. In the pet world, molecules are scaled to 1/10th the normal size and represented by pet atoms, which require only 2% of the original atom number. This allows for efficient collision detection to generate tight packings as well as large-scale coarse-grained molecular dynamics (MD) simulations, for example, to insert the HIV RNA into its capsid core.

The methods described here are fully automated and built on a collection of open-source YASARA macros, which can easily be adapted by users for their own needs. In addition, an infrastructure for sharing these macros and the generated mesoscale models has been set up at www.YASARA.org/petworld.

## Results and Discussion

### Creating a Coarse-Grained Representation—the Pet World

Mesoscale modeling inherently depends on a coarse-grained representation of the molecules to maintain the required computations tractable. Unfortunately, there are three specific needs that do not match today’s well-developed methods for coarse-grained simulation: first, the models are so large that representations with up to five beads per amino acid like the Martini force field^10^ are computationally still too expensive. Second, visualizing the results as all-atom models is only feasible if the GPU can use a technique called instancing, where each type of molecule is present only once in the GPU memory, and identical copies are drawn at all occurrences of this molecule. This implies that any simulation done before should deliberately not try to capture internal dynamics (like an elastic network model^11^) but instead minimize conformational changes. Third, the representation of large molecules such as proteins should be more fine-grained on the surface than on the inside, so that a tight packing of coarse-grained representations does not lead to clashes when switching back to all-atom visualization.

**Figure 1:**
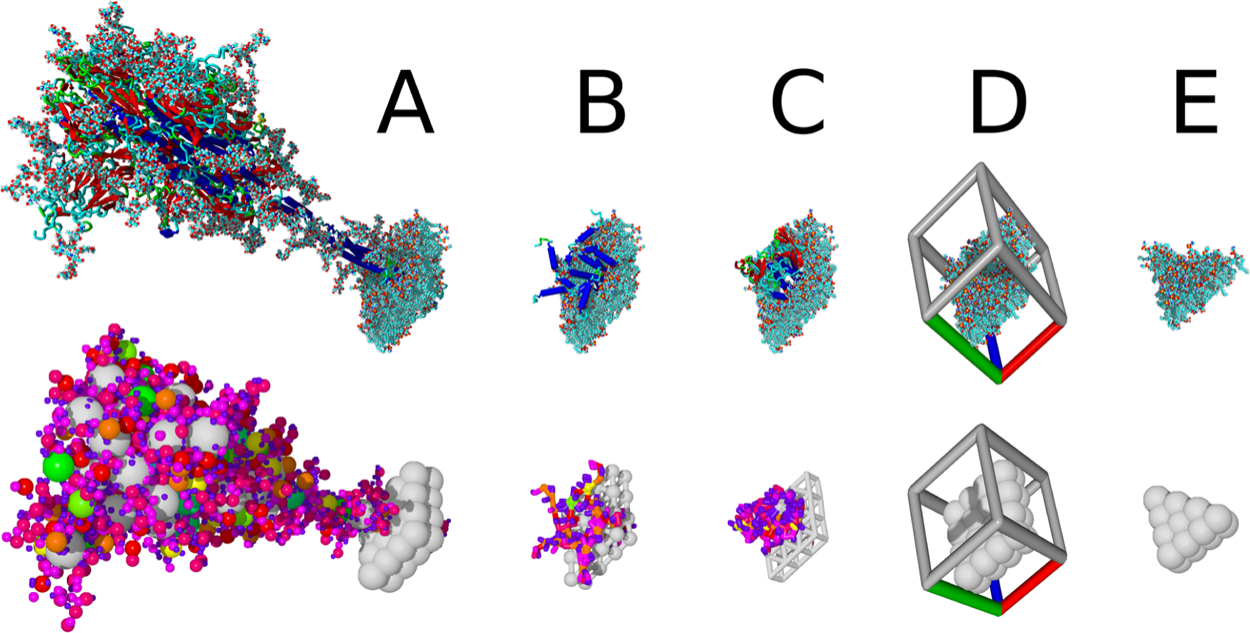
Five building blocks needed to construct a model of the SARS-CoV-2 envelope, all-atom based models on top, and the corresponding pet molecules at the bottom [scaled up for comparability, pet atoms are colored by size from blue (0.2 Å) via orange to cyan (1.1 Å) and gray (1.2 Å)]: the automatic embedding procedure starts with the detection of transmembrane helices and strands, places the predicted transmembrane regions inside rhombic membrane blocks, and runs a short MD simulation so that the interactions between the membrane and protein can stabilize. During the simulation, the lipids close to the periodic cell boundaries are kept fixed, so that opposite edges of all building blocks match each other. (A) Spike protein block,^14^ (B) E-protein block,^15^ (C) M-protein block,^15^ (D) empty membrane block with the rhombic simulation cell used for equilibration, and (E) triangular block to close the leftover holes. To demonstrate that pet atoms form a large number of bonds (up to 25 for large pet atoms in the core of a protein), pet molecules in B and C are shown as ball-and-sticks and sticks, respectively.

To address these challenges, our algorithm to create a coarse-grained structure starts with a density grid of the molecule and places spheres in two passes. First to optimally trace the surface and second to fill the remaining holes (see Materials and Methods). Additionally, we decided to shrink the coarse-grained representation by a factor of 10 (hence the name “pet”) to aid 3D visualization and simulation: close to the eye, depth perception is better, and Z-buffer resolution is higher. If no details are left, it makes little sense to keep objects far away. Also, optimized MD simulation algorithms tend to harmonize better with atoms that are not exceptionally large. We defined 12 pet atoms, artificial chemical elements with symbols “O1” to “O9”, “Oa”, “Ob”, “Oc”, and van der Waals (vdW) radii ranging from 0.1 to 1.2 Å in steps of 0.1 Å. These correspond to spheres with real-world radii of 0.1–1.2 nm. The name “pet atom” was also chosen because molecular modeling software can handle them most efficiently by adding them to the list of chemical elements and then many functions including building, visualization, and input/output in file formats like PDB or mmCIF are readily available. Some exemplary pet molecules are shown in Figure 1. Overlapping pet atoms are connected with bonds (i.e., harmonic springs) to keep their initial distance during a simulation; large pet atoms in the core of a protein sometimes form up to 25 bonds (Figure 1B,C). Phospholipid bilayers and nucleic acids are shrunk in a way that optimally preserves their structure (see Materials and Methods).

### Assembling Giant Structures from Pet Molecules

For mesoscale systems, it is impractical to store all atoms explicitly because their enormous number would quickly exhaust the available memory. We use two approaches to compress the data: assembly is done with pet molecules, which reduces the atom count by a factor of 50, and all-atom visualization uses GPU instancing as described above, which reduces the memory requirements by a factor of 40–1000. Instancing implies that one first needs to create building blocks and then join instances (i.e., identical copies) of these blocks to construct the final model, just like Lego bricks.^12^

A tricky part of mesoscale models are phospholipid membranes with embedded proteins. Due to the membranes’ arbitrary shape, there is normally no exact way to construct them with instances of the identical building blocks. Fortunately, approximate solutions work well in practice. We define the shape of the membrane with a triangle mesh, which can be created manually using 3D modeling software like Blender, or algorithmically, for example, by subdividing an icosahedron to obtain a sphere mesh. The mesh triangles are chosen to be as far as possible equilateral and of the same size, for example, with the help of the Instant Meshes tool.^13^ Then, two neighboring triangles are joined to rhombi with angles of 60 and 120° (named “ideal rhombus”). Finally, these rhombi are filled with instances of rhombic membrane blocks, with and without transmembrane proteins (Figure 1).

The various membrane blocks are created by all-atom MD simulation in rhombic simulation cells with periodic boundaries (Figure 1D). Most MD packages support simulation in triclinic cells and can thus deal with the 90/60/90° angles of rhombic simulation cells, which fit the ideal mesh rhombus. Membrane proteins are inserted into the rhombic blocks by scanning them for transmembrane α-helices, β-barrels, or lipid anchors and placing the corresponding hydrophobic surface region inside the membrane. Then, the mesh can be plastered with rhombic membrane blocks whose neighboring edges fit together correctly without bumps between phospholipid molecules, thanks to the periodic boundaries used during the simulation. For every non-trivial mesh, there are some locations where two rhombi touch with incorrect edges, and where triangular blocks (Figure 1E) are needed to close the holes between rhombi, but this is hardly noticeable in practice.

As soon as the membranes have been built, pet DNA/RNA is created directly from a FASTA sequence file. The remaining space is filled by creating a neighbor search grid and placing the pet proteins at random locations with random orientations, rejecting those that bump into the existing pet atoms or are outside their compartment, as defined by the membrane polygon mesh used above.

### MD Simulation of the Pet World

**Figure 2:**
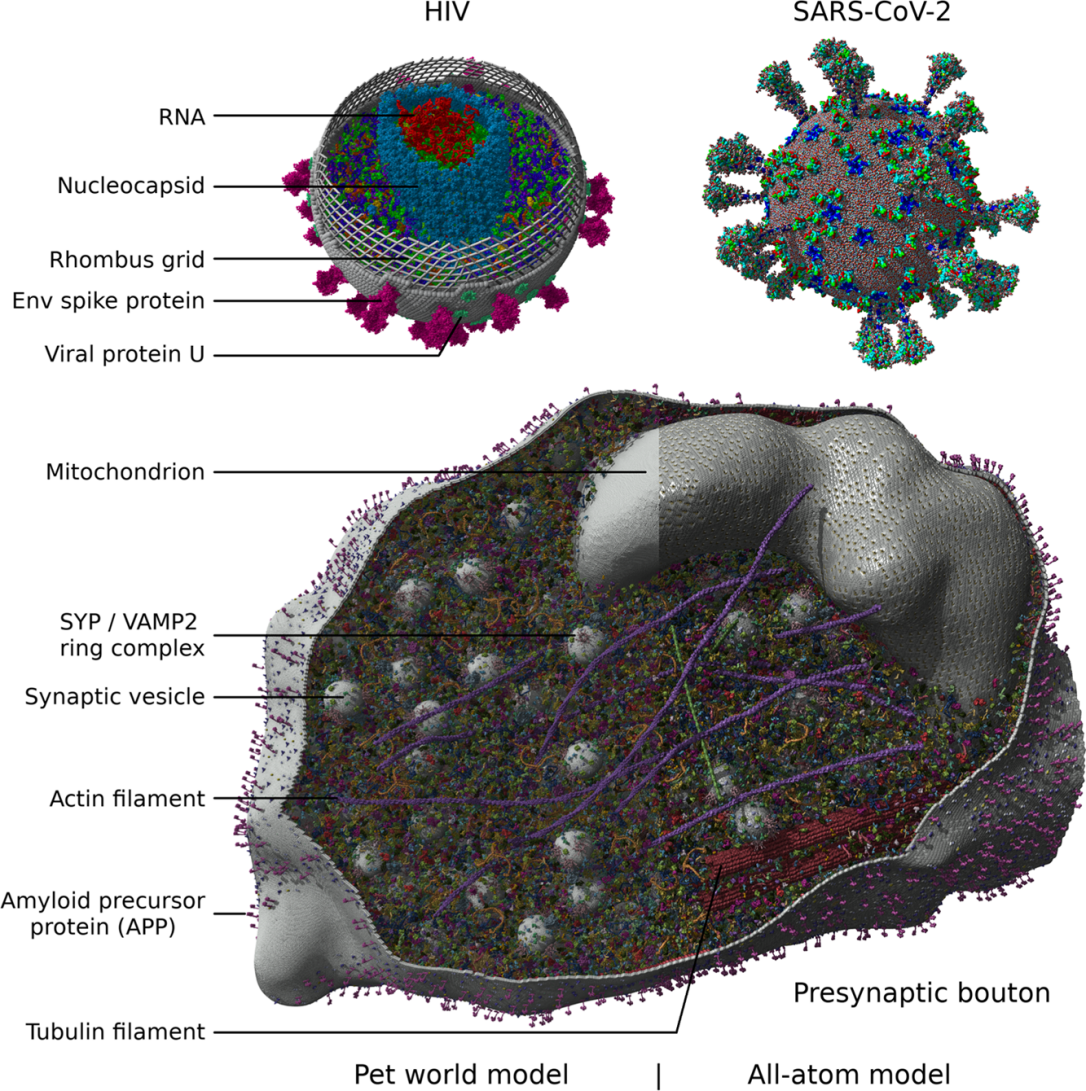
Molecular models of HIV (top left), SARS-CoV-2 (top right), and a presynaptic bouton (bottom center). For comparison, the left half of the figure shows coarse-grained pet models, while all-atom models are displayed in the right half. The 1:10 scaling factor has been removed for clarity. For HIV, a part of the rhombus mesh to be filled with rhombic membrane blocks is shown.

Having assembled a scene of pet molecules, it is often helpful to explore the time dimension of the system, which can be done efficiently using MD simulations. For example, to resolve clashes when creating very densely packed systems, to explore mesoscale motions, to simulate packing mechanisms (like the injection of a self-assembling nucleocapsid into a budding particle), or to improve the realism of the initial assembled system (e.g., if a somewhat artificial packing algorithm was used, like for packing the two RNA strands of HIV into the capsid core, as shown in Figure 2).

The first step is to assign force field parameters. We chose the same equations for describing interactions between pet atoms as used for the AMBER force field,^16^ since these are already implemented in YASARA.^9^ The normal way of defining atom types and their equilibrium intraaction parameters (bond lengths, angles, and dihedrals) is not possible, since pet atoms are placed at random locations with random bond lengths between them. Therefore, we simply define 12 atom types for the 12 pet elements and then assign equilibrium bond lengths, angles, and dihedrals based on the atom coordinates at the start of the simulation. The pet molecules need to be as stiff as possible, so that they do not deviate too much from the identical instances used for visualization in the real world. Also, multi-domain proteins connected by flexible peptide linkers must not undergo significant internal conformational changes, or clashes could arise after reverting back to an all-atom model. If the goal was not visualization but simulation (i.e., if the system was small enough to be in reach of MD supercomputers), then such conformational changes would be postponed to the actual simulation. The same mass of 8 u is assigned to all pet atoms to maximize the possible time-step, which depends on the mass of the lightest atom.

For the actual simulation, we found that a multiple time-step of 0.5 fs (bonded intraactions) and 1 fs (non-bonded interactions) is required for stable simulations due to the small size and tight coupling of the pet atoms. As cutoff, 3 Å proved sufficient to handle the mostly repulsive packing forces in this work (i.e., the largest pet atoms with 1.2 Å radius could feel each others’ vdW attraction if the gap between them was smaller than 0.6 Å). An implicit solvent model with long-range electrostatics would be needed for simulations on a longer timescale and to handle for example DNA association of positively charged proteins realistically.

### There and Back Again—Expanding Pet World Models

Having finished the model building and refinement, the pet models can be viewed either directly or expanded back to full all-atom details. During creation of pet molecules, a backup of the original all-atom molecule is stored. Upon expansion, the backup is restored and one instance is created for each pet molecule copy. At this point, we transition from one compression method (coarse-grained pet models) to another (GPU instancing of all-atom models). For free nucleic acids, no all-atom structures are available at this step, since they have been built as pet molecules. For their expansion, short nucleic acid fragments are superposed on the pet atoms to obtain full coordinates.

### MD Simulation of the Real World

If the system happens to be small enough that not only interactive visualization but also a MD simulation on a supercomputer can be envisioned, then some additional processing of the membranes is needed. As mentioned before, every membrane (except flat isolated pieces) has imperfections, that is, rhombi that are somewhat distorted and smaller than an ideal rhombus. Lipids at the edges of these rhombi clash into their neighbors, which is hardly visible, but a problem for MD simulation, so these lipids need to be deleted. In regions of high membrane curvature, lipids need to be transferred from one side to the other to ensure equal density of both lipid layers.

### Three Modeling Examples

As a practical application of the methods described in the previous sections, we built molecular models of SARS-CoV-2,^17^ HIV,^4^ and the presynaptic bouton^18^ (Figure 2).

The envelope of the SARS-CoV-2 model consists of the three embedded membrane proteins E, M, and S (Figure 1) and has been filled with hexon (“eggs-in-a-nest”) and tetrahedron (“pyramid”) ribonucleoprotein oligomers;^17^ the actual RNA will be added later as soon as more experimental data become available. The coarse-grained pet model consists of 175,622 pet atoms and the expanded all-atom model of 14.2 million atoms instanced from 221,542 unique atoms (Figure 2, top right). An interactive movie describing the whole model building process by showing how to construct the SARS-CoV-2 virion has been uploaded to www.YASARA.org/movies.

The HIV virion model is partly based on a CellPack recipe,^4^ contains two embedded and two membrane-associated proteins in the envelope, and carries two RNA strands inside the capsid core particle, coupled at the dimerization initiation sequence. The HIV capsid core particle content (RNA and proteins) was relaxed using coarse-grained MD simulation. Including lumen-filling proteins, there are 752,019 pet atoms (Figure 2, top left). After expansion, the entire particle consists of 39.7 million atoms, instanced from 581,520 unique atoms.

Finally, the largest and most complex model is the presynaptic bouton, which is based on protein structures and a membrane mesh described before.^18^ The pet model’s 16 million atoms expand to a 3.6 billion all-atom model, instanced from 2 million unique atoms and 51 proteins, including protein filaments, endosomes, the mitochondrial envelope, and 307 synaptic vesicles. The synaptic vesicles are instanced from a single vesicle model, which is made available as a separate entry in the PetWorld database. The filaments inside the bouton were grown through repeated stacking of segment instances with small random orientation variations. As described in the following section, we used color schematics for instances far away from the view plane to help distinguish between different proteins as well as membrane lipids. For comparison, pet atoms have been colored the same way (Figure 2, bottom center).

We would like to encourage the reader to contribute a model to the growing PetWorld database and are happy to help, for example, with membrane meshes. Especially, molecular modeling classes are a good occasion, since students can take responsibility of modeling individual proteins and then join their efforts to assemble the complete scene.

### Molecular Graphics with Reduced Levels of Detail for Distant Objects

**Figure 3:**
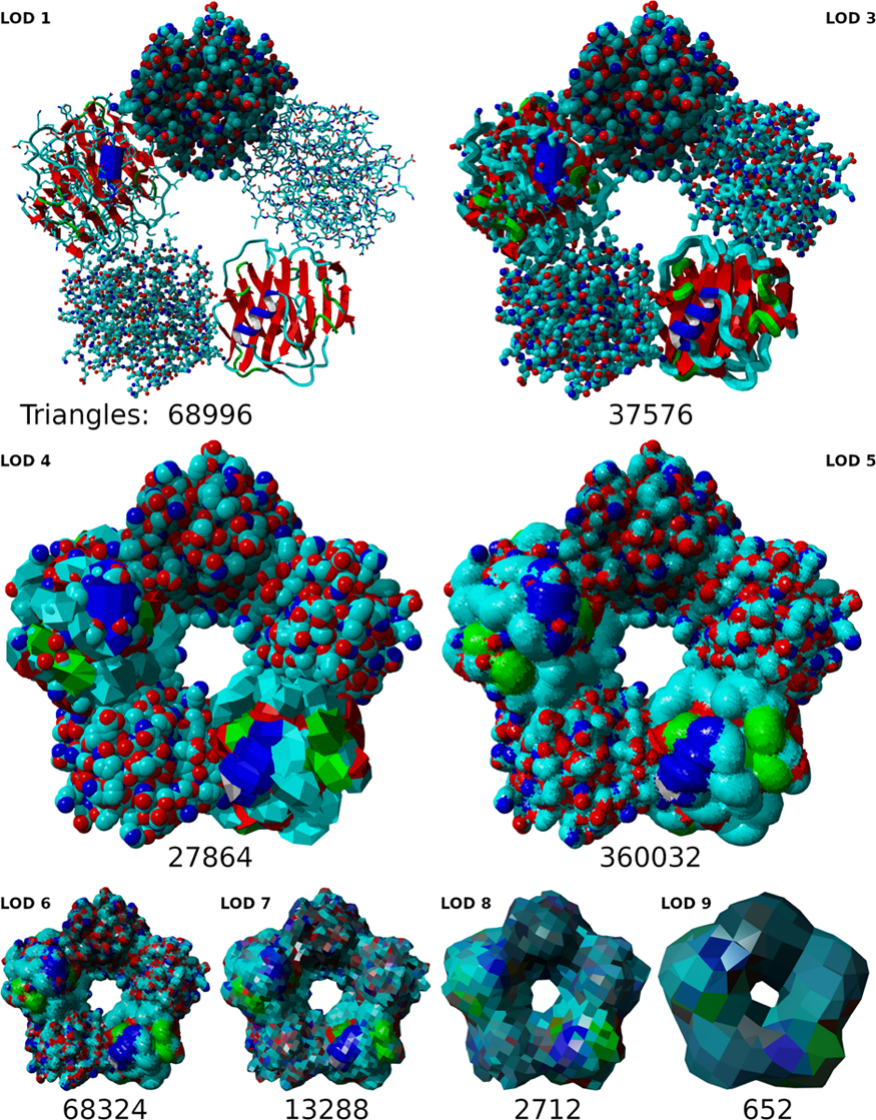
LODs with total triangle count for five atom and secondary structure styles of pentameric human serum amyloid P component (PDB-ID: 1SAC). In clockwise order: balls, sticks, ribbon, ball-and-sticks, and cartoon with stick side chains. The pentamer is shown with full details (LOD 1), at an intermediate distance (LODs 3–4) and far from the view plane (LODs 5–9). LOD 2 is almost the same as LOD 1 and omitted for brevity. Flat shading was used to increase polygon visibility. Individual atoms are darkened according to ambient occlusion, pre-calculated per object by the CPU.^8^ Additional full-scene ambient occlusion is calculated by the GPU with the SAO approach.^21^ The transition from ball and stick impostors to a grid-density-based isosurface happens at LOD 5, which is shown in a small distance region only due to its enormous polygon count.

While the atom count in pet models is small enough to allow immediate visualization, a lot more efforts are needed to display the expanded all-atom models at interactive frame rates. Biomolecules are commonly represented by space-filling balls, balls connected with sticks or plain sticks, in conjunction with cartoon cylinders, ribbons, tubes, and arrows to visually convey information about common protein secondary structures like α-helices and β-sheets, as shown in Figure 3.

When viewing structures containing thousands of proteins, most of them will be far away from the view plane with details invisible to the human eye, but would remain computationally costly if rendered at full detail. The implementation of distance-dependent discrete levels of detail (LODs) allows us to drastically reduce the graphical workload without any noticeable loss of visual quality.

Le Muzic et al.^19^ described an approach where groups of atom balls are replaced with single larger balls at growing distance. Since this works for balls only and requires to fade the individual atom colors to one single “far color” per protein to hide the popping artifacts, we went a different route. Our graphics engine splits the reduced LOD representation of distant objects into three stages:

(1) Near the view plane, ball and stick impostors are drawn with two triangles per ball/stick (since Vulkan does not support quads).^20^ Two LODs are used for protein secondary structure meshes (LOD 1, the full detail representation is shown in Figure 3, LOD 2 uses only four instead of eight mesh segments per amino acid residue).
(2) At intermediate distance, the engine slowly increases ball and stick radii with growing distance from the view plane and shifts secondary structure mesh vertices outward along their normals in the vertex shader. The purpose of this model inflation is to fill the intermediary empty space and obtain compact objects ready for transition to the third stage and it also helps the user to recognize smaller objects at larger distances. The outward shift creates gaps between the four sides of the rectangular β-strands as well as the top and bottom caps of helix cylinders, which are bridged with additional polygons, creating octagonal and conical shapes. In addition, two lower resolution secondary structure LODs are used: LOD 3 reduces the vertices in each mesh segment from 12 to 6, and LOD 4 uses only two instead of four mesh segments per amino acid, as shown in Figure 3.
(3) At far distance, the engine switches to a different representation and each compacted object is replaced by a single surface mesh (Figure 3, LOD 5). Each mesh is an isosurface extracted from a density grid representing the protein, incorporating the current atom and secondary structure style. Atoms drawn as sticks are at this point indistinguishable from balls and treated as such. At set distances, the current surface is replaced by one constructed from a reduced density grid, resulting in coarser meshes with fewer vertices (Figure 3, LODs 6–9). LOD 5 surfaces are shown only in a small sector of the view volume as they need at least 10 times as many triangles as LOD 4 and are therefore computationally very expensive. The advantage is that LOD 5 allows for a smooth transition without obvious popping artifacts when switching from the representation style used in LODs 1-4 to the single surface mesh style of LODs 5–9.

### Drawing Billions of Atoms with the Vulkan Graphics API

**Figure 4:**
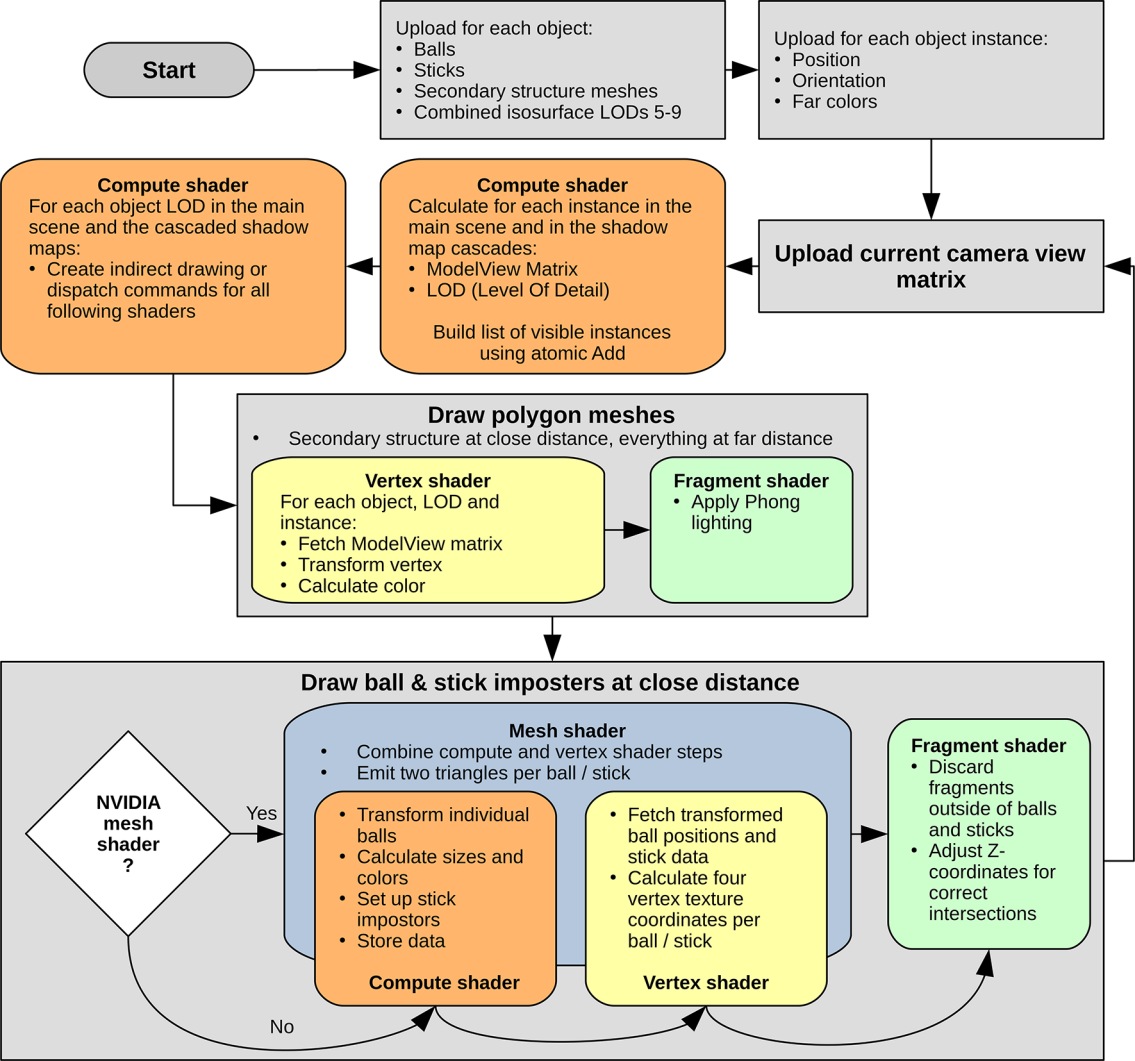
Flowchart of the Vulkan graphics API pipeline for the drawing of balls, sticks, and polygon meshes, including protein secondary structure. Rounded rectangles denote GPU tasks. The compute shader preprocesses each object in the main scene and up to three cascaded shadow maps^21,22^ by calculating the ModelView matrix and LOD for each instance, after which indirect drawing and dispatch commands for the following shaders are created. While the protein secondary structure and the distant LODs 5–9 are simple polygon meshes (Figure 3) drawn with Phong lighting, the ball and stick impostors at closer distances are drawn in two steps: first, their eye space coordinates and parameters (color, radius) are calculated by a compute shader and temporarily written to global memory, from where a vertex shader reads them to calculate four corner vertices per impostor. Second, the fragment shader discards pixels outside the ball or stick and adjusts the Z-coordinate to yield correct intersections between impostors as described previously.^20^ NVIDIA’s mesh shader, introduced in 2018 with the Turing architecture, combines the compute and vertex shader steps in a single mesh shader and avoids some quadruplicated calculations in the vertex shader. We hoped for a measurable performance increase, but got only 1% for drawing balls. At least it helps to save GPU storage memory for the compute shader output.

Vulkan is a high-performance 3D graphics API developed by the Khronos Group and first released in 2016. Close to metal flexibility and control over modern GPU functionality and memory coupled with low overhead and precompiled shaders sets Vulkan on the path of becoming the successor of OpenGL for high-performance cross-platform graphics. A workflow chart of our graphics pipeline is shown in Figure 4.

**Figure 5:**
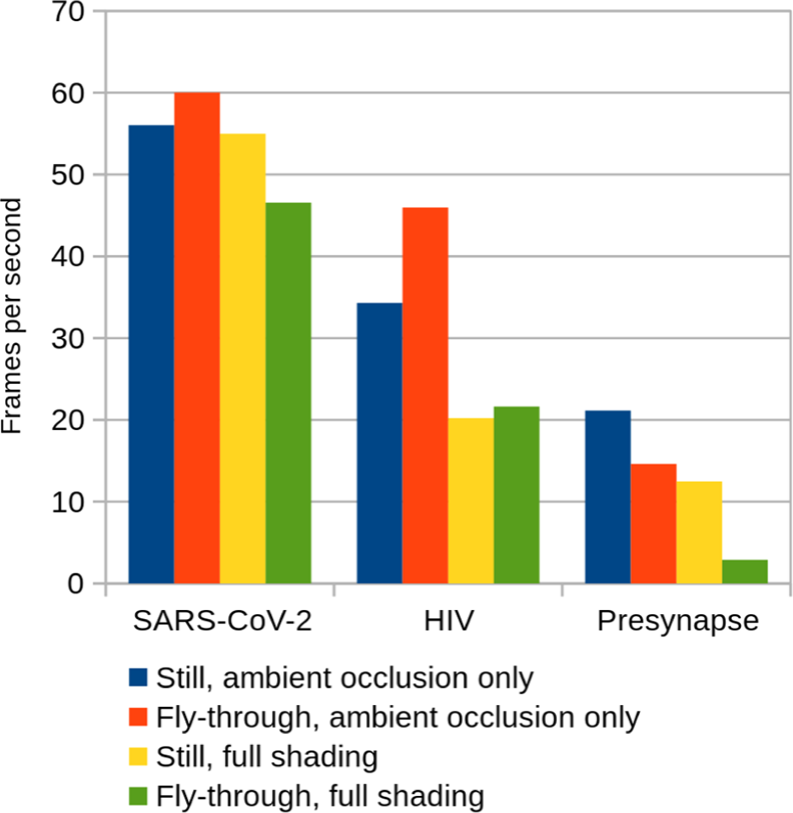
Frames per second at Full HD resolution 1920 × 1080 using a GeForce RTX 2080 graphics card with and without cascaded shadow maps, for all three models displayed either as a screen-filling still scene or as a dynamic fly-through (average fps shown). Results are capped at the screen refresh rate of 60 Hz.

The Vulkan API is fast enough to enable YASARA to interactively visualize biomolecular systems with billions of atoms in all common styles from balls and sticks to secondary structure ribbons on consumer-grade hardware through instancing of modular building blocks generated as described above. The frame rates achieved on a GeForce RTX 2080 graphics card are shown in Figure 5. Only the presynaptic bouton is so large that the frame rate drops below convenient levels and shadows have to be disabled on this card, relying on ambient occlusion and fog only to aid depth perception. Significantly faster graphics cards have already been released.

## Materials and Methods

### Creation of Pet Molecules—the Shrinking Algorithm

The first step of the algorithm is to scale the target molecule down to 1/10th of the original size, then approximate the shape of the molecule by spheres called pet atoms, and lastly add covalent bonds between neighboring pet atoms. A set of twelve pet atom sizes is used, with radii ranging from 0.1 to 1.2 Å in steps of 0.1 Å. Nucleic acids and lipid bilayer membranes are treated as special cases described later.

**Figure 6:**
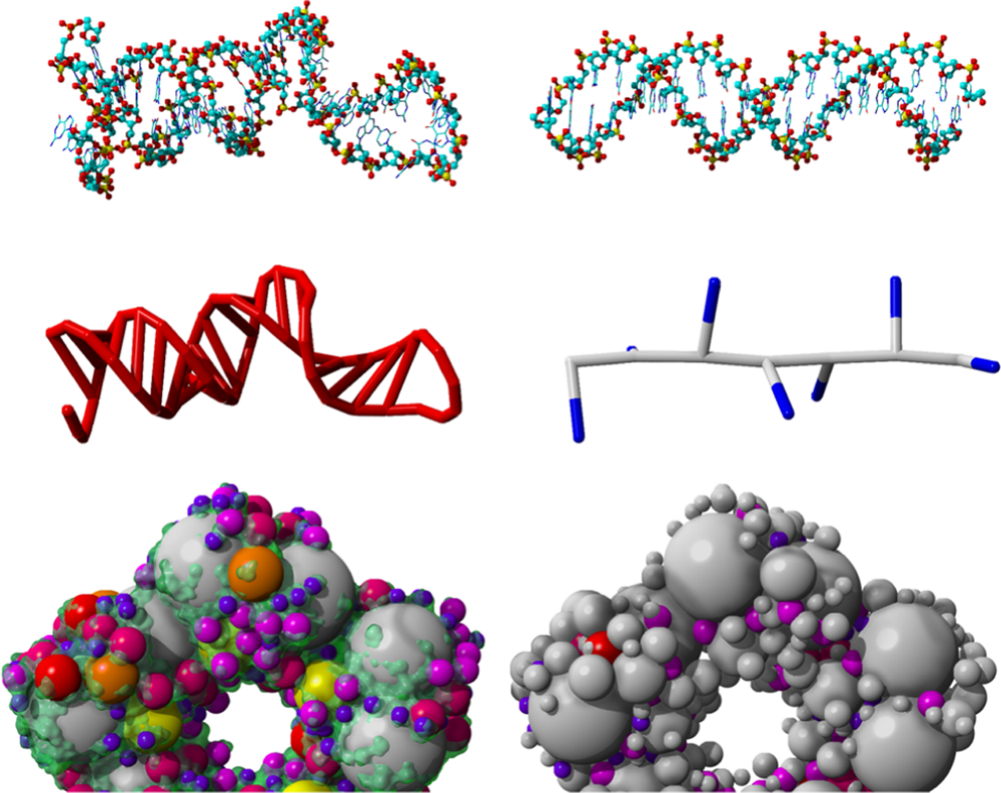
Top: all-atom representation of a single-stranded RNA hairpin loop (left) and a double-stranded DNA fragment. Center: pet atom representation, styled as sticks, of the RNA (left) and DNA (right) fragments shown on top. Each nucleotide of a single-stranded nucleic acid is replaced by a 0.5 Å pet atom (red); each triplet of double-stranded nucleic acid residue pair is replaced by one 1.2 Å pet atom (white) together with a 0.1 Å pet atom (blue) tracking the helix backbone. Additional bonds between the paired pet atoms preserve the RNA secondary structure during MD simulations. Bottom: Pet versions of molecules like the pentameric human serum amyloid P component (PDB-ID: 1SAC) are created in two passes. First, the molecular surface (green) is traced with appropriately sized pet atoms (left). In the second pass (right), remaining holes inside the molecule left by pet atoms in the first pass (gray) are filled with additional pet atoms (colored).

The all-atom model of the target molecule is mapped to a cubic density grid with a cube side length of 2^13+*n*^ fm, with n taking the values 1, 2, or 3 if the radius of the smallest used pet atom is 0.1, 0.2, or 0.3 Å. A good performance/accuracy trade-off is to use pet atoms down to radius 0.2 Å, as shown in Figure 1, and then the grid spacing is 0.328 Å. The density grid stores two flags at each grid point; the first indicates if the grid point lies inside or outside of the molecule, and the second is set when a pet atom has been placed that incorporates the point. The grid is filled in two passes with pet atoms in order of descending size. The goal of the first pass is to approximate the surface shape of the density grid. Holes on the inside will be closed in the second pass (Figure 6, bottom). A pet atom probe of the current size is moved along the grid and scored at each position according to the current pass
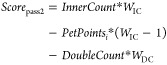
1
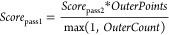
2where *InnerCount* is the number of grid points inside the pet atom that are also flagged as inside the molecule. *PetPoints* is the total number of points encompassed by the current pet atom with size *i*. The number of points already accounted for by other accepted pet atoms is given by *DoubleCount*. The weight values are chosen as *W*_IC_ = 3.5 and *W*_DC_ = 1.5. *OuterPoints* is the number of points in a two-point wide shell around the pet atom and *OuterCount* is the number of these points flagged as inside the molecule.

If the score exceeds a pass-dependent minimum score, the pet atom is considered for placement, and the current pet atom position, score, and size are stored in a table.
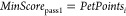
3
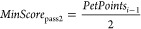
4

The minimum first pass score is equal to the maximum second pass score, which according to eq 1 is simply *PetPoints*, since the best score is obtained if *InnerCount* equals *PetPoints* and *DoubleCount* is zero. The minimum second pass score of pet atom *i* is half of the second pass maximum score of the next smaller pet atom *i* – 1. For the smallest pet atom—which does not have a smaller one—it is set to *PetPoints*/2. To speed up the score computation, only the change of the score is calculated as the pet atom probe is moved along the *X*-axis of the grid, adding the points that newly enter the probe on one side and subtracting those that leave the probe on the other side.

When the sweep with a pet atom probe of a given radius is finished, the table of scored positions is sorted. Pet atoms are placed starting with the highest-scoring pet atom. After rescoring to consider the effect of pet atoms placed before (which may cause the current atoms to be sorted down in the table or deleted if the score dropped below the minimum score), all grid points enclosed by the pet atom are flagged as covered and the pet atom is added to the growing molecule.

In the end, pet atoms with a distance smaller than the sum of their vdW radii are joined with a chemical bond to exclude them from intermolecular vdW and electrostatic forces during a simulation and to keep the structure stable. Residual pet atoms without bonds are bonded to the closest atom. The pet atoms and their bonds are mapped to a graph to search for disconnected clusters and join them with additional bonds to prevent the molecule from falling apart.

### Automatic Embedding of Transmembrane Proteins

To identify the transmembrane region in a known protein structure, several approaches have been developed, for example, the TMDET server.^23^ We use a straightforward approach that searches for secondary structure elements that only have exposed (= side-chain surface > 20% of maximum) hydrophobic residues (Ala, Val, Leu, Ile, Met, Phe, Tyr, and Trp) on one side, while not showing strongly polar residues (Asn, Gln, Lys, Arg, Asp, and Glu). For β-strands, “side” is obvious; for α-helices, “side” is one-third of the helix circumference (120°) in any direction. A transmembrane strand is 7–14 residues long, with >3 exposed hydrophobic residues. A transmembrane helix is 12–29 residues long, with >4 exposed hydrophobic amino acids. A regression line is fit through the Cα atoms of each identified transmembrane element, and the protein is oriented such that the sum of all line directions is normal to the membrane. Finally, the protein is scanned along this normal vector to find the region with the largest number of exposed hydrophobic residues. Those that are also part of transmembrane segments identified above count as five residues.

A quadratic membrane block, equilibrated using all-atom MD with periodic boundary conditions, is used as the basic building block to create a rhombic membrane block of a given size. Lipids are mutated in place to reach any requested composition of phosphatidylethanolamine, phosphatidylcholine, and phosphatidylserine, or simply replaced with cholesterol if needed. Copies of the quadratic membrane blocks are joined against each other to reach a size large enough for the chosen rhombus dimensions, followed by the deletion of lipids to reach the target lipid density and approximate rhombic shape. An MD simulation in a successively shrinking rhombic simulation cell with periodic boundaries compresses the lipids to the exact desired rhombic membrane shape. This is done in vacuo, with the membrane stabilized by forces on lipid heads and tails to ensure correct alignment. A solvation and equilibration phase concludes the creation of the empty membrane block (Figure 1D). Additionally, an equilateral triangular block is produced by splitting the rhombus in half (Figure 1E) and used later to fill the remaining holes between rhombi.

All the membrane proteins are embedded in the same membrane block (to make sure that the edges match), using the orientation normal to the membrane calculated above. Membrane proteins that occur very often can be embedded multiple times with random rotation and lateral shift, so that the final result does not look suspiciously regular. During the embedding, the protein is temporarily truncated 5 Å above and below the membrane, so that large external parts (Figure 1A) can extend beyond the size of a membrane rhombus. If the protein’s part in the membrane plane exceeds the size of a single rhombus, *N* × *N* rhombi are joined to form a super-rhombus. Next, the protein is placed inside the membrane and overlapping lipids are deleted (first only strongly bumping ones with interaction energy larger than 10 pJ, and after energy minimization, the remaining ones that got trapped inside the protein and could not escape). The embedding process is finalized by a simulation in explicit solvent for equilibration, which makes sure that the lipids smoothly cover the protein. Lipids at the cell boundary are fixed in place during equilibration to guarantee a seamless fit between the membrane blocks of all proteins. Membrane blocks that are used to tessellate small regular spheres like a synaptic vesicle are bent slightly for a better fit.

### Creation of Pet Membranes

When shrinking a molecule, membrane phospholipids and nucleic acids are not fed to the algorithm described above, but handled differently. At the beginning of the shrinking process, if a considerable amount of phospholipids is detected, the given object is classified as a membrane block. The lipids are separated from any present transmembrane proteins and mapped onto their own density grid. While the protein molecules undergo shrinking as described above, the lipid density grid is analyzed to determine the membrane block size and shape (rectangle, rhombus, or triangle). Knowing the size and shape of the block, pet atoms with a radius of 1.2 Å are systematically positioned in two layers to approximate the bilayer membrane. In the end, the pet counterparts of the block transmembrane proteins and lipid bilayer membrane are fused to a single pet molecule (Figure 1).

### Creation of Pet Nucleic Acids

Analogous to lipid membranes, nucleic acids are handled separately. Single-stranded DNA/RNA is replaced by a chain of pet atoms with a radius of 0.5 Å, one for each nucleotide, placed at the position of the C1* atom (Figure 6, top/center left). Double-stranded nucleic acids are identified by screening for two strands that are sequential in the structure, have the same length, and are anti-parallel with all pairs of C1* atoms closer than 1.2 Å (on a pet scale). A coarser model is used because double-stranded DNA has less structural variability and is typically much longer. Each triplet of nucleotide pairs is represented by a 1.2 Å pet atom, placed on the helical axis at the middle base pair (Figure 6, top/center right). A second 0.1 Å helper atom is placed 1.2 Å away from the main atom and thus the nucleic acid’s axis to track the helical backbone (see section about force field parameter assignment).

### Creation of Pet Genomes

While the pet versions of proteins and their bound nucleic acid fragments can be obtained by shrinking all-atom structures obtained from the PDB or by structure prediction, this is not possible for long nucleic acids, like mRNAs or entire genomes. Instead, these are built from pet atoms using their sequence provided in FASTA format, optionally with a secondary structure assignment in dot-bracket notation (for single-stranded nucleic acids). The algorithm first extracts the nucleic acid sequences present in the binding protein structures shrunk before and then scans the provided FASTA sequence for all occurrences—these become the protein-binding sites. If a certain binding site is present more than once, the respective number of copies of the binding protein is created. Finally, the genome is assembled by joining stretches of newly built pet nucleic acids with the pet nucleic acid fragments present in the binding protein structures. The resulting conformation is currently random, but a lattice model could be applied if experimental data about the genome structure were available.^24,25^

### Tessellation of Spheres and Other Shapes with Membrane Blocks

As described in the Results and Discussion section, the shape of membranes is defined using meshes of near-equilateral triangles, which are paired to rhombi and then filled with rhombic building blocks (Figure 1). In general, surfaces (notably spheres) cannot be tessellated by exactly equilateral triangles. Furthermore, the plastering of these triangular meshes with oriented rhombi is neither unambiguous nor without triangular gaps. Hence, a specific tessellation must be picked. First of all, each mesh triangle is rated by looping over its three neighbors with which a rhombus can be formed and scored according to the size and shape compared to the “ideal rhombus” with angles of 60 and 120°
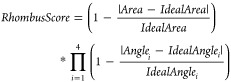
5

The deviation in size and shape compared to the ideal rhombus is quantified by the product of the differences in rhombus area and the four corner angles.

After picking the triangle with the highest *RhombusScore* sum as the starting point (each triangle has three scores, since it can participate in three rhombi), three tessellations are generated corresponding to these three starting rhombi. Iteratively, the four neighboring rhombi (excluding those already visited before) are scored and added to a sorted list, from which the top scoring rhombus is picked to continue the tessellation. The algorithm stops when no rhombus can be placed and only triangular holes are left. The total score is given by the sum of rhombus scores and the list with the highest total score is chosen as the final tessellation. The top 90% in the list are randomly shuffled to ensure a uniform distribution; then, membrane blocks with embedded proteins are placed consecutively at the listed rhombi, skipping rhombi that lead to collisions between proteins. In the end, the leftover rhombi and triangular holes are filled with empty membrane blocks.

### Force Field Parameter Assignment for Pet Molecules

To run MD simulations of pet molecules, we chose the AMBER force field equation. There are 12 new atom types named o1–o9, oa, ob, and oc with vdW radii from 0.1 to 1.2 Å and a mass of 8 u (Tables S1 and S2 in Supporting Information). The default bond stretching force constant is 575 kcal/(mol·Å^2^), the angle bending force constant is 300 kcal/(mol·rad^2^), and the dihedral barrier (pk) is 5 kcal/mol. The charge of each pet atom is simply the sum of all atom charges it represents, scaled with 0.1 (to yield electrostatic forces that match the scaled size).

Force field parameter assignment needs to differentiate between two different kinds of atoms:

First, pet atoms that have been obtained by shrinking known structures. Their coordinates are known; they are identified by assigning them a *B*-factor of 0, and their equilibrium bond lengths, angles, and dihedrals can simply be taken from the coordinates at the start of the simulation. Since bonds are added between all pet atoms that are closer than the sum of their vdW radii, large central atoms can have up to two dozen bonds. Consequently, the normal AMBER procedure to assign angle terms to allbound atom triplets and dihedral terms to allbound atom quartets would be too costly. As an optimization, no angle terms are added within 3-rings (since these are uniquely determined by the three bonds anyway), only two neighboring angle terms are added within 4-rings (which is also enough to define their geometry), and dihedral terms are skipped unless absolutely needed. Due to the dense network of bonds, most dihedral terms are superfluous, but we found that a good recipe is to add a dihedral term to atoms A–B–C–D if A forms less than four bonds and B forms two bonds; the parameters are idiv = 1, pn = 1, and phase = current dihedral + π. We also exclude all angle terms with equilibrium values <20 or >160° (because the force calculation has singularities at 0 and 180°) and dihedral terms that involve atom triplets with excluded angle terms.

Second, pet atoms that represent free nucleic acids (alone or bridging protein-binding sites) and have been built from the FASTA sequence file. Their initial coordinates are only crude approximations. These atoms are assigned a *B*-factor > 0, and as soon as one of these atoms is part of a bond, angle, or dihedral, the equilibrium value is not taken from the current coordinates but from Table 1. As described above, double-stranded nucleic acids are represented by a string of pet atom pairs with 1.2 and 0.1 Å radii (Figure 6) that represent three nucleotide pairs each. Angular forces have singularities at 0 and 180°, so the equilibrium angle of 180° between the gray atoms needs to be enforced by two gray–gray–blue angles of 90° each. The blue–gray–gray–blue dihedrals are set to 3× the twist in Table 1 to keep track of the location of the DNA backbone and make sure that continuous all-atom representations can be calculated at joining points with binding proteins.

**Table 1:** Default Parameters Used to Build Nucleic Acids and to Assign Force Field Parameters

default parameters for nucleic acids		
	B-DNA	A-RNA
α	–46.85	–73.728
β	–146.06	–173.666
γ	36.41	48.878
ε	155	–171.286
ζ	–95.18	–66.929
rise per base pair	3.365 Å	2.87768 Å
shift between complementary strands (accounts for tilted bases)	0.5 Å	–2 Å
twist, rotation per base pair	36	36.787
distance of C1* from main axis	6.05 Å	7.318 Å
sequential C1*–C1* distance	4.95 Å	5.330 Å
C1*–C1_paired_^*^ distance	10.8 Å	12.68 Å
C1*–C1*–C1* angle	153.795	150.108
C1*–C1_paired_^*^–C1*3′ angle	54.581	51.234
C1*–C1_paired_^*^–C1*5′ angle	100.424	105.066
C1*–C1*–C1*–C1* dihedral	24.907	19.717

Some values differ a bit from those published elsewhere because they depend on the geometry of the individual nucleotides joined (included in PDB format in Table S3 in Supporting Information) and have been tuned to yield correct hydrogen bonding also for longer fragments. C1* is the C1 atom of the ribose ring, where a pet atom with radius 0.5 Å is placed to represent one nucleotide, C1*–C1_paired_^*^–C1*3′ is the angle formed by a C1*, its paired C1*, and the 3′ neighbor of the paired C1*.

### Expansion of Pet Molecules to Full Atomic Detail

During creation of pet molecules, a backup of the original all-atom molecule is stored. Upon expansion, the backup is restored and one instance is created for each pet molecule copy. The distances between instances are enlarged by a factor of 10 to retain relative positions and moved along the *Z*-axis to keep the projected size the same. A backup of the pet molecule is stored in the expanded molecule data structure to ensure that the scene can be shrunken and expanded instantly and repeatedly in place.

### Expansion of Pet Genomes to Full Atomic Detail

While proteins and their bound nucleic acid fragments can be expanded easily to the known structure used to create the pet version, this is not possible for all the remaining nucleic acid fragments, which have been created directly as pet atoms as described above.

For double-stranded genomes, which consist of two pet atoms per three nucleotide pairs, the expansion is rather simple since the structure is very regular. It is enough to build a linear dsDNA or dsRNA (using parameters from Table 1 and nucleotide coordinates from Table S3 in the Supporting Information), bend it to follow the trace of the large 1.2 Å pet atoms, and twist it to keep the backbone in sync with the direction indicated by the small 0.1 Å pet atoms.

For single-stranded genomes, which are built from one pet atom per nucleotide located at the C1* position, two methods are employed: if nucleotides form a double helix locally, a double-helical fragment with three base pairs in standard conformation (see above) is superposed on six C1* atoms, and the coordinates of the middle base pair are used. For unpaired nucleotides, a local coordinate system centered on the C1* is created. The *X*-axis is the sum of the vectors to the next and the previous C1*, the *Z*-axis is the cross product of the *X*-axis and the vector to the next C1*, and the *Y*-axis is the cross product of *Z*- and *X*-axes. Then, the positions of the base’s N3 and C6 atoms are calculated using their known coordinates in this local coordinate system (provided in Table S4 in Supporting Information). N3 and C6 are chosen because they occur in all nucleotide bases. Then, a nucleotide with default conformation is superposed onto the three atoms C1*, N3, and C6. Finally, if the distance between the O3* of the previous nucleotide and the P of the current nucleotide is too large, the size of the gap is minimized by adjusting the three dihedral angles C3*–C4*–C5*–O5*, C4*–C5*–O5*–P, and C5*–O5*–P–O3*_prev_ using the cyclic coordinate descent algorithm from robotics.^26^

### Storing Pet and Expanded Models in mmCIF Format

Models consist of objects (proteins, membrane blocks, and nucleic acids, possibly consisting of multiple chains/molecules). Each model can be saved in the mmCIF file format to enable straightforward cross-platform data sharing. Each object is stored as a PDB “model” (not to be confused with the entire model) with increasing atom_site.PDB_model_num. Pet models are saved atom by atom without instances and thus without compression, using chemical element symbols O1–O9, OA, OB, and OC, which should be displayed as spheres with radii from 0.1 to 1.2 Å. All-atom models use object instances and are compressed by storing them as biological assemblies, which means that only one transformation matrix (i.e., one line in the mmCIF file) is needed per instance (_pdbx_struct_oper_list.matrix/.vector). To make sure that each _pdbx_struct_assembly_gen entry can reference a certain object, _pdbx_struct_assembly_gen.PDB_model_num items were added as a little extension. Also, the mmCIF format does not normally allow to give a separate name to each object (= PDB model), that is why we added _pdbx_model entries that provide the object/model name (_pdbx_model.name) and the number of instances (_pdbx_model.instances). If the parser encounters a model with more than one instance, it creates them immediately from the pdbx_struct_assembly data. Otherwise, the biological assembly data are only read, but ignored until the user explicitly requests an oligomerization.

### Reduced LODs via Density Grids and Isosurface Extraction

As described above, for LOD 4 and higher, we extract isosurface meshes from a density grid via the Marching cubes algorithm,^27^ which necessitates the creation of a grid representation of secondary structure meshes and ball impostors (the inflated sticks are indistinguishable from balls and therefore treated as such). Representing molecules by a density grid allows creating lower resolution LODs easily by averaging densities over eight neighboring grid cells.

While adding spherical density for individual atoms to the grid is essentially trivial, the protein secondary structure is harder to convert to a grid density. Our algorithm works as follows: each ungapped residue stretch is characterized by a spline connecting either the Cα atoms (loops, helix ribbons) or interpolated points near the Cα atoms (β-strands, helix cylinders). The spline is further subdivided into four segments between two spline points (i.e., between two amino acid residues). We follow the spline from segment to segment and fill the grid with a single combined value representing the density and surface color at each grid point.
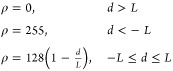
6The density ρ is the signed distance *d* from the secondary structure surface to the current grid point, clamped to a range of [−*L*, *L*] and mapped to a single byte [0, 255]. *L* is the length of one grid cube, so the density drops linearly from the maximum value of 255 one grid cube length inside the surface to a minimum value of 0 at one grid cube length outside the surface. The isosurface is extracted at density 128, corresponding to surface distance 0.

The grid-filling algorithm starts out with the grid point nearest to the beginning of the residue spline and iteratively adds all neighboring grid points to the list of next to be processed grid points if the calculated density of the current grid point is larger than zero, flood-filling the grid. An auxiliary grid keeps track to which segment each grid point belongs to, as grid points can fall into multiple segments because of surface self-intersections or overlaps with subsequent residue stretches. In these cases, we re-evaluate the density and keep the highest density value and corresponding surface color for each grid point.

**Figure 7:**
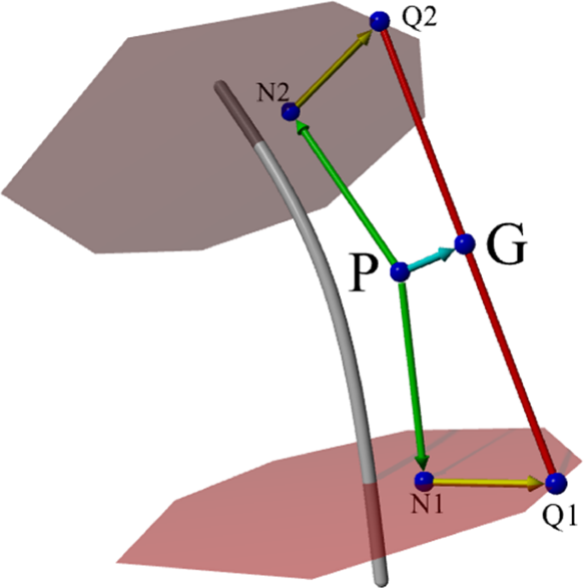
Grid point P is projected (green) onto the upper and lower cut planes of the current spline segment. In each plane, we determine the ray (yellow) through the projected point N normal to the closest edge of the surface cross section to find the piercing point Q. Point G is given through normal projection (cyan) of P onto the line joining the piercing points (red). The distance between P and G serves as an estimate of the distance from P to the actual mesh surface.

Each segment is capped by an upper and lower cut plane orthogonal to the spline. The actual surface is approximated by linear interpolation between cut plane cross sections. To find the distance of a grid point to the approximated surface, we first project the grid point onto each cut plane to determine in each plane the ray running through the projected grid point normal to the surface, then pin down the intersection point of the ray with the surface cross section. Finally, the distance from the grid point to the connecting line between the piercing points serves as an estimation of the distance to the actual surface (Figure 7).

The spline tangent vector is normal to the cut planes, which ensures that the cross sections of the visualization surface have always the same shapes: circles for tubes and helix cylinders, and irregular octagons with *p*4 symmetry for arrows and ribbons. The *p*4 symmetry octagon equates to a symmetrically cut rectangle and is uniquely defined by two side lengths and their distances to the center. Piercing points on circles are trivially given through their respective radii, and for octagons, the intersection of the ray with the nearest pierced side is determined trigonometrically.

## Data and Software Availability

An implementation is available as part of the YASARA molecular modeling and simulation program from www.YASARA.org. The free YASARA View program includes the described Vulkan graphics engine and can be used to explore the presented models, which can be downloaded from www.YASARA.org/petworld, a Creative Commons platform for sharing giant biomolecular structures. The packing and MD algorithms are part of YASARA Dynamics. The open source YASARA macro to build each structure is included in the PetWorld database entry, additional function libraries can be found at www.YASARA.org/md_library.mcr and www.YASARA.org/pack_library.mcr, and Python syntax highlighting is recommended.

## Supporting Information Available

AMBER atom types and masses; AMBER Lennard-Jones parameters; default DNA and RNA nucleotide geometries; and reference positions of the N3 and C6 atoms in each nucleotide (TXT)

## References

[ref1] Amaro R. E.; Mulholland A. J. Multiscale Methods in Drug Design Bridge Chemical and Biological Complexity in the Search for Cures. Nat. Rev. Chem. 2018, 2, 0148 10.1038/s41570-018-0148. 30949587 PMC6445369

[ref2] Marrink S. J.; Corradi V.; Souza P. C. T.; Ingólfsson H. I.; Tieleman D. P.; Sansom M. S. P. Computational Modeling of Realistic Cell Membranes. Chem. Rev. 2019, 119, 6184–6226. 10.1021/acs.chemrev.8b00460. 30623647 PMC6509646

[ref3] Goodsell D. S. Inside a Living Cell. Trends Biochem. Sci. 1991, 16, 203–206. 10.1016/0968-0004(91)90083-8. 1891800

[ref4] Johnson G. T.; Autin L.; Al-Alusi M.; Goodsell D. S.; Sanner M. F.; Olson A. J. cellPACK: A Virtual Mesoscope to Model and Visualize Structural Systems Biology. Nat. Methods 2015, 12, 85–91. 10.1038/nmeth.3204. 25437435 PMC4281296

[ref5] Mindek P.; Kouril D.; Sorger J.; Toloudis DD.; Lyons B.; Johnson G.; Groller M. E.; Viola I. Visualization Multi-Pipeline for Communicating Biology. IEEE Trans. Vis. Comput. Graph. 2018, 24, 883–892. 10.1109/TVCG.2017.2744518. 28866552

[ref6] Klein T.; Autin L.; Kozlíková B.; Goodsell D. S.; Olson A.; Gröller M. E.; Viola I. Instant Construction and Visualization of Crowded Biological Environments. IEEE Trans. Vis. Comput. Graph. 2018, 24, 862–872. 10.1109/TVCG.2017.2744258. 28866533 PMC5746312

[ref7] Krieger E.; Koraimann G.; Vriend G. Increasing the Precision of Comparative Models with YASARA NOVA - A Self-Parameterizing Force Field. Proteins: Struct., Funct., Bioinf. 2002, 47, 393–402. 10.1002/prot.10104. 11948792

[ref8] Krieger E.; Vriend G. YASARA View - Molecular Graphics for All Devices - From Smartphones to Workstations. Bioinformatics 2014, 30, 2981–2982. 10.1093/bioinformatics/btu426. 24996895 PMC4184264

[ref9] Krieger E.; Vriend G. New Ways to Boost Molecular Dynamics Simulations. J. Comput. Chem. 2015, 36, 996–1007. 10.1002/jcc.23899. 25824339 PMC6680170

[ref10] Marrink S. J.; Risselada H. J.; Yefimov S.; Tieleman D. P.; de Vries A. H. The MARTINI Force Field: Coarse Grained Model For Biomolecular Simulations. J. Phys. Chem. B 2007, 111, 7812–7824. 10.1021/jp071097f. 17569554

[ref11] Hu G.; Di Paola L.; Liang Z.; Giuliani A. Comparative Study of Elastic Network Model and Protein Contact Network for Protein Complexes: The Hemoglobin Case. BioMed Res. Int. 2017, 2017, 1–15. 10.1155/2017/2483264. PMC5294226 28243596

[ref12] Le Muzic M.; Autin L.; Parulek J.; Viola I. cellVIEW: A Tool for Illustrative and Multi-Scale Rendering of Large Biomolecular Datasets. Eurographics Workshop Vis. Comput. Biomed. 2015, 2015, 61 10.2312/vcbm.20151209. 29291131 PMC5747374

[ref13] Jakob W.; Tarini M.; Panozzo D.; Sorkine-Hornung O. Instant Field-Aligned Meshes. ACM Trans. Graph. 2015, 34, 1–15. 10.1145/2816795.2818078.

[ref14] Woo H.; Park S.-J.; Kyo Choi Y.; Park T.; Tanveer M.; Cao Y.; Kern N. R.; Lee J.; Yeom M. S.; Croll T. I.; Seok C.; Im W. Developing a Fully Glycosylated Full-Length SARS-CoV-2 Spike Protein Model in a Viral Membrane. J. Phys. Chem. B 2020, 124, 7128–7137. 10.1101/2020.05.20.103325. 32559081 PMC7341691

[ref15] Heo L.; Feig M. Modeling of Severe Acute Respiratory Syndrome Coronavirus 2 (SARS-CoV-2) Proteins by Machine Learning and Physics-Based Refinement. bioRxiv 2020, 10.1101/2020.03.25.008904.

[ref16] Maier J. A.; Martinez C.; Kasavajhala K.; Wickstrom L.; Hauser K. E.; Simmerling C. ff14SB: Improving the Accuracy of Protein Side Chain and Backbone Parameters from ff99SB. J. Chem. Theory Comput. 2015, 11, 3696–3713. 10.1021/acs.jctc.5b00255. 26574453 PMC4821407

[ref17] Yao H.; Song Y.; Chen Y.; Wu N.; Xu J.; Sun C.; Zhang J.; Weng T.; Zhang Z.; Wu Z.; Cheng L.; Shi D.; Lu X.; Lei J.; Crispin M.; Shi Y.; Li L.; Li S. Molecular Architecture of the SARS-CoV-2 Virus. Cell 2020, 183, 730–738. 10.1016/j.cell.2020.09.018. 32979942 PMC7474903

[ref18] Wilhelm B. G.; Mandad S.; Truckenbrodt S.; Krohnert K.; Schäfer C.; Rammner B.; Koo S. J.; Classen G. A.; Krauss M.; Haucke V.; Urlaub H.; Rizzoli S. O. Composition of Isolated Synaptic Boutons Reveals the Amounts of Vesicle Trafficking Proteins. Science 2014, 344, 1023–1028. 10.1126/science.1252884. 24876496

[ref19] Le Muzic M.; Parulek J.; Stavrum A. K.; Viola I. Illustrative Visualization of Molecular Reactions Using Omniscient Intelligence and Passive Agents. Comput. Graph. Forum 2014, 33, 141–150. 10.1111/cgf.12370.

[ref20] Tarini M.; Cignoni P.; Montani C. Ambient Occlusion and Edge Cueing to Enhance Real Time Molecular Visualization. IEEE Trans. Visual. Comput. Graph. 2006, 12, 1237–1244. 10.1109/tvcg.2006.115. 17080857

[ref21] McGuire M.; Mara M.; Luebke D. Scalable Ambient Obscurance. Proceedings of ACM SIGGRAPH/Eurographics High-Performance Graphics 2012, 2012; Vol. 12, pp 97–103.

[ref22] Engel W. Cascaded Shadow Maps, Shader X5: Advanced Rendering Techniques; Charles River Media, 2007; pp 197–206.

[ref23] Tusnády G. E.; Dosztányi Z.; Simon I. TMDET: Web Server for Detecting Transmembrane Regions of Proteins by Using their 3D Coordinates. Bioinformatics 2005, 21, 1276–1277. 10.1093/bioinformatics/bti121. 15539454

[ref24] Johnson G. T.; Goodsell D. S.; Autin L.; Forli S.; Sanner M. F.; Olson A. J. 3D Molecular Models of Whole HIV-1 Virions Generated with cellPACK. Faraday Discuss. R. Soc. Chem. 2014, 169, 23–44. 10.1039/c4fd00017j. PMC4569901 25253262

[ref25] Goodsell D. S.; Jewett A.; Olson A. J.; Forli S. Integrative Modeling of the HIV-1 Ribonucleoprotein Complex. PLoS Comput. Biol. 2019, 15, e1007150 10.1371/journal.pcbi.1007150. 31194731 PMC6592547

[ref26] Canutescu A. A.; Dunbrack R. L. Jr. Cyclic Coordinate Descent: A Robotics Algorithm for Protein Loop Closure. Protein Sci. 2003, 12, 963–972. 10.1110/ps.0242703. 12717019 PMC2323867

[ref27] Lorensen W. E.; Cline H. E. Marching Cubes: A High Resolution 3D Surface Construction Algorithm. Comput. Graph. 1987, 87, 163–169. 10.1145/37401.37422.

